# Non-Coding RNAs Regulate Spontaneous Abortion: A Global Network and System Perspective

**DOI:** 10.3390/ijms23084214

**Published:** 2022-04-11

**Authors:** Jianyu Gan, Ting Gu, Huaqiang Yang, Zheng Ao, Gengyuan Cai, Linjun Hong, Zhenfang Wu

**Affiliations:** 1 National Engineering Research Center for Breeding Swine Industry, College of Animal Science, South China Agricultural University, Guangzhou 510642, China; jygan@stu.scau.edu.cn (J.G.); tinggu@scau.edu.cn (T.G.); yangh@scau.edu.cn (H.Y.); cgy0415@scau.edu.cn (G.C.); 2 Key Laboratory of Animal Genetics, Breeding and Reproduction in the Plateau Mountainous Region, Ministry of Education, College of Animal Science, Guizhou University, Guiyang 550025, China; zao@gzu.edu.cn; 3 Guangdong Provincial Key Laboratory of Agro-Animal Genomics and Molecular Breeding, College of Animal Science, South China Agricultural University, Guangzhou 510642, China; 4 State Key Laboratory for Conservation and Utilization of Subtropical Agro-Bioresources, Guangzhou 510642, China

**Keywords:** spontaneous abortion, miRNA, lncRNA, circRNA, competing endogenous RNA, systematic network

## Abstract

Spontaneous abortion is a common pregnancy complication that negatively impacts women’s health and commercial pig production. It has been demonstrated that non-coding RNA (ncRNA) is involved in SA by affecting cell proliferation, invasion, apoptosis, epithelial-mesenchymal transformation (EMT), migration, and immune response. Over the last decade, research on ncRNAs in SA has primarily concentrated on micro RNAs (miRNAs), long non-coding RNAs (lncRNAs), and circular RNAs (circRNAs). In this review, we discuss recent ncRNA studies focused on the function and mechanism of miRNAs, lncRNAs, and circRNAs in regulating SA. Meanwhile, we suggest that a ceRNA regulatory network exists in the onset and development of SA. A deeper understanding of this network will accelerate the process of the quest for potential RNA markers for SA diagnosis and treatment.

## 1. Introduction

Spontaneous abortion (SA) is clinically defined as the loss of pregnancy before 24 weeks of gestation without any external intervention [1,2]. Two or more consecutive SA in the first 20 weeks are defined as recurrent spontaneous abortions (RSA) [3]. SA not only has a negative impact on women’s health and reproduction but also poses a substantial risk to global commercial pig production (about 20–30% of pig embryos develop SA during 12–30 days of pregnancy) [4]. It is estimated that approximately 5% of women of childbearing age have RSA [5], which causes psychological distress and has a negative impact on their welfare. Understanding the pathogenesis of SA and its treatment are critical for both human reproductive health and commercial porcine production. It has recently been determined that endocrine, immune, environmental, infectious, and genetic factors all play a role in SA development [6,7]. Nevertheless, about half of patients cannot be identified as having the pathogenesis of SA (defined as unexplained recurrent spontaneous abortion (URSA)), posing significant challenges in treating SA. URSA remains a substantial challenge in obstetrics due to a lack of safe and effective treatment options and reliable early detection tools [8]. Hence, studies and research that can explain the mechanism of SA are required.

Non-coding RNAs (ncRNAs) are RNAs that do not encode proteins and include but are not limited to small nuclear RNA (snRNA), small nucleolar RNA (snoRNA), ribosomal RNA (rRNA), transfer RNA (tRNA), PIWI-interacting RNA (piRNA), circular RNA (circRNA), long non-coding RNA (lncRNA), and microRNA (miRNA). ncRNAs play essential biological roles even though they do not encode proteins. For instance, they can regulate a wide range of paramount life activities by participating in chromosomal remodeling, gene transcription, and post-transcriptional modification [9]. Over the last decade, research on ncRNAs in SA has primarily concentrated on miRNAs, lncRNAs, and circRNAs.

Herein, we discuss recent ncRNA studies focused on the function and mechanism of miRNAs, lncRNAs, and circRNAs in regulating SA. Since the competing endogenous RNA (ceRNA) network hypothesis was proposed in 2011, researchers have become increasingly aware of the possible potential application of ceRNA network regulation in diagnosing and treating a wide range of diseases [10]. Based on the findings of these studies on pregnant diseases [11,12,13,14,15], we hypothesize that there may be a broad and complex ceRNA regulatory network that will induce SA when network regulation is disrupted. We anticipate that our viewpoint will be helpful as a reference and will aid future research into the pathogenesis of SA and the search for potential RNA markers for diagnosis and treatment.

## 2. Pathogenesis of SA

**Figure 1:**
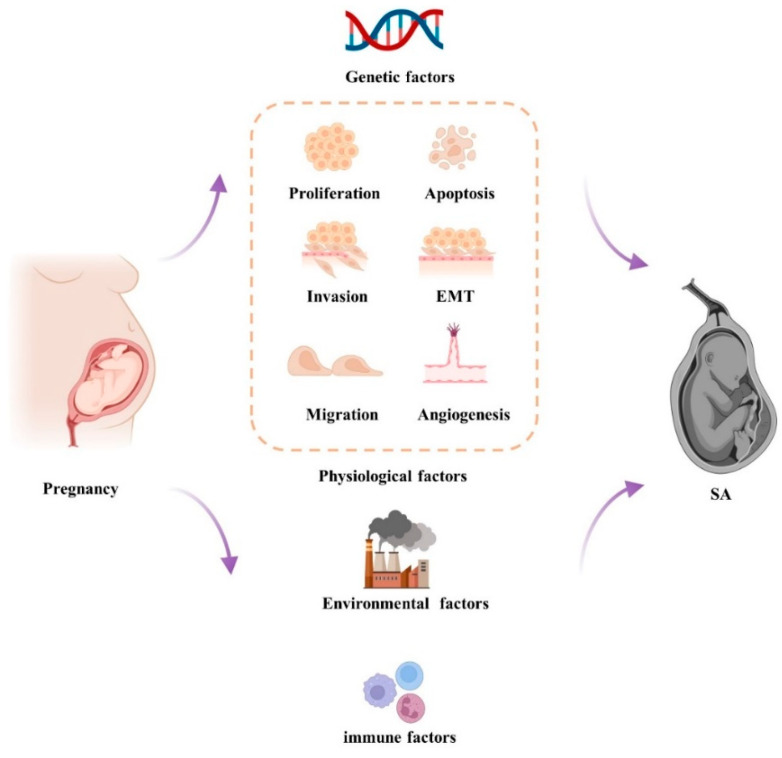
Etiology of SA. It is currently known that the factors affecting SA are genetic factors, physiological factors, environmental factors, and immune factors. Among them, genetic factors are the most crucial etiology of SA. EMT, epithelial-mesenchymal transformation.

There are different pathogenesis of SA, including genetic factors [16], physiological factors [17], immune factors [18], and environmental factors [19] (Figure 1). Genetic factors are considered the main contributors to sporadic SA and RSA due to fetal chromosome abnormalities accounting for 50–60% of cases [2]. However, about 40–50% of RSA patients demonstrate normal karyotypes, and the etiologies are still unclear, which is thought to have URSA [20,21,22]. In the early stage of normal pregnancy, the correct performance of the functions of placental trophoblast cells directly affects the survival of embryos. In humans and mice, cytotrophoblast cells differentiate into syncytiotrophoblast and extravillous trophoblast (EVT) cells after implantation [23]. Subsequently, EVT cells invade the maternal endometrium, fixing the placenta to the uterine wall and leading to the remodeling of the maternal spiral artery to provide nutrition for the developing fetus [23]. Any abnormal step in this process may lead to placenta-related pathological pregnancy, including SA [24].

In recent years, with the increasing research of ncRNAs in SA, researchers have a more thorough understanding of the mechanisms of ncRNAs regulating SA. Indeed, miRNAs [25,26], lncRNAs [27,28,29], and circRNAs [30,31] are all involved in the modulation of the pathogenesis of SA. Afterward, researchers also found some ncRNAs that can be regarded as markers of SA, which play a critical role in the diagnosis of SA. However, regarding how these ncRNAs participate in the regulation of SA, the specific mechanism is still unclear, resulting in great limitations in the use of these ncRNAs in clinical diagnosis, treatment, and porcine production.

## 3. miRNAs and Spontaneous Abortion

### 3.1. Overview of miRNAs

miRNAs is an endogenous, short, non-coding, and highly conserved calss of molecule that play a role in the mechanism of post-transcriptional gene expression by inhibiting the translation of protein-encoded genes or cutting target mRNAs [32,33]. The first miRNA, LIN-4, was found in Caenorhabditis elegans in 1993 [34]. Afterward, studies on miRNA have emerged continuously and even become a research hotspot. miRNAs can bind to the mRNAs of target downstream genes, affecting the stability and transcription of the target mRNAs. A special feature of miRNAs is that one kind of miRNA can regulate the expression of several kinds of mRNAs, while one kind of mRNA can also be targeted by multiple kinds of miRNAs, which means that a small number of miRNAs can regulate about 60% of the protein-coding genes [17,35]. Over 1000 kinds of miRNAs have been revealed to participate in modulating complex processes in vivo, such as cellular growth, differentiation, immune response, and tissue remodeling, and some of them play a critical role in multiple diseases states, including SA [36,37]. As a result, some miRNAs in the maternal circulatory system have been screened as good biomarkers for monitoring the progression of normal pregnancy and the existence of pregnant diseases, which can be used to prevent and treat some reproductive diseases [38].

### 3.2. miRNAs Affect SA by Regulating Apoptosis

In 1998, Kokawa et al. [39] first proposed that apoptosis balance disruption could be one of the primary causes of SA. Studies confirmed that some miRNAs could indeed regulate apoptosis by affecting different pathways, thus triggering or inhibiting SA. For instance, Liu et al. [40] discovered that miR-93 expression was increased in the chorionic villi of patients with RSA, and the expression of targeted BCL2-like 2 (*BCL2L2*) disrupted the balance of apoptosis, cell proliferation, migration, and invasion and promoted the occurrence of SA.

In addition, miR-19b and miR-494 can regulate the expression of phosphatase and tensin homolog (*PTEN*) in a synergistic manner, influencing SA occurrence [41]. *PTEN* is a well-known tumor suppressor gene that regulates apoptosis in various cells and is involved in the cell cycle, growth, migration, and death through the PI3K/AKT signaling pathway [42]. RSA occurrence can be effectively prevented with miR-19b upregulation and miR-494 downregulation, which inhibits cell apoptosis through the expression of their common target gene *PTEN* [41].

Furthermore, miRNA-365, an upstream regulator of *MDM2*/*P53* expression, cell cycle progression, and apoptosis in trophoblast cells, was discovered to regulate trophoblast apoptosis through serum/glucocorticoid regulatory kinase 1 (SGK1) [43]. Since *SGK1* is the direct target gene of miR-365, silencing *SGK1* can cause trophoblast cell cycle arrest and apoptosis, and *SGK1* overexpression can reduce the effect of miR-365 on apoptosis and *MDM2*/*P53* expression [43]. Both these mechanisms have a direct bearing on SA. Furthermore, when Dong et al. [44] investigated the role of miRNA-199B-5p and SGK1 in pregnancy maintenance, they discovered that miR-199B-5p expression was significantly upregulated in the aborted decidua, while miR-199B-5p overexpression in human endometrial stromal cells and transgenic mice reduced *SGK1* expression, and the in vitro and in vivo results were consistent and comparable. Therefore, miR-199b-5p, like miR-365, may control apoptosis by targeting *SGK1*. Another study revealed that by targeting poly (ADP- ribose) polymerase 1 (*PARP1*), miR-520 could facilitate trophoblast apoptosis induced by DNA damage, thereby contributing to the onset and progression of RSA [45].

### 3.3. miRNAs Are Key Regulators in the Physiological Processes of the Placenta

SA is directly linked to placental physiological processes of invasion, migration, EMT, and angiogenesis. When these processes are hampered and overburdened, uterine-placental dysfunction occurs, increasing the risk of SA [46]. The Wnt signaling pathway governs numerous cellular functions, including migration and invasion, and can be regulated by ubiquitin-specific peptidase 25 (USP25) [47]. Ding et al. [48] found that miR-27A-3p can regulate the expression of *USP25* by binding to the 3′ untranslated region of *USP25* in trophoblast cells, thus regulating the migration and invasion of EVT and trophoblast cells. EMT, an evolutionarily conserved developmental program, has been linked to carcinogenesis and confers metastatic properties on cancer cells. When miR-27A-3p was transfected with microRNA mimics or inhibitors, the expression of *USP25* in RSA’s patients’ placental villi was down-regulated, and the processes of EMT, invasion, and migration were inhibited [48].

Furthermore, miR-16 can regulate placental angiogenesis and development by regulating vascular endothelial growth factor (*VEGF*) expression and participating in the pathogenesis of RSA [49]. VEGF plays a pivotal role in regulating the invasion and proliferation of trophoblast cells. When the VEGF signal pathway is inhibited, it may suppress trophoblast cell proliferation and invasion, resulting in SA [50,51]. The vascular endothelial growth factor receptor-2 (VEGFR2) is the receptor for VEGF and contributes to the proliferation, migration, and invasion of trophoblasts by binding to core proteins [52]. MiR-219a can inhibit the function of VEGFR2 and its downstream PI3K/AKT/NF-κB signal pathway by targeting the expression of *VEGFR2*, which inhibits the excessive proliferation and invasion of trophoblast cells by negative regulation [26]. Meanwhile, Zhang et al. [25] identified that miR-155-5p regulated SA by activating the NF-κB signaling pathway. MiR-155-5p overexpression can reduce the release of inflammatory cytokines such as IL-6, IFN-γ, TNF-α, and IL-10 by decidual stromal cells and inhibit the apoptosis of decidual stromal cells.

### 3.4. miRNAs Participate in the Regulation of Immunity

In early pregnancy, it is necessary to experience the processes of placental development and spiral artery remodeling, which require the participation of immune cells and immune molecules; thus, SA is largely related to the imbalance of the maternal immune system [53,54,55]. Natural killer (NK) cells and macrophages surround the spiral artery, while interstitial EVT cells are recruited by NK cells and macrophages to replace vascular endothelial cells in the spiral artery thus initiating the process of remodeling [55,56]. Considering the indispensable role of NK cells and macrophages, many scholars have studied miRNAs and their targets on NK cells and M1 macrophages in patients with SA.

In the NK cells of decidual tissue of patients with URSA, Li et al. [57] studied the expression levels of six kinds of miRNA in decidual NK cells of URSA patients and revealed that miR-34a-3p/5p, miR-141-3p/5p, and miR-24 might link to URSA. These miRNAs target *P53*, *PTEN*, and IFN-γ (*IFNG*), respectively, to regulate apoptosis, proliferation, and angiogenesis [58,59,60,61]. Considering the effects of P53, IFNG, and PTEN on placental villi, trophoblast invasion, migration, EMT, and apoptosis have been discussed previously, it is reasonable to deem that these miRNAs in NK cells of URSA patients play a fundamental role in regulating SA.

Moreover, Li et al. [62] used miRNA microarray to study the miRNAs map of NK cells in decidual tissues of patients with URSA. Once again, it was found that there was a close relationship between abnormal expression of miRNAs and URSA, and 50 differentially expressed miRNAs were identified to construct miRNA-Gene-Network, miRNA-Go-Network, and miRNA-Gene-TF-Network. In miRNA-Go-Network, the key miRNA is hsa-miR-5787, which inhibits cell growth by targeting eukaryotic translation initiation factor 5 (*EIF5*) [63]. From the results of GO analysis of down-regulated miRNAs, the Wnt receptor signal pathway is a key functional pathway, and the prediction of transcription factors indicates that Krüppel-like factor 6 (*KLF6*, also known as *CPBP*) may be the key gene regulating these genes and miRNAs network [62].

M1 macrophages also play a crucial role in placental immunity and spiral artery remodeling. MiR-103 can negatively regulate the polarization of M1 macrophages by inhibiting the STAT1/IRF1 signal pathway, resulting in inhibiting the occurrence of SA. Overexpressed miR-103 can effectively reduce the absorption and M1 macrophages polarization of mouse embryos. Therefore, when miR-103 is reduced, it is possible to induce RSA by promoting the STAT1/IRF1 signaling pathway to increase the polarization of M1 macrophages. Moreover, miR-103 can be used to distinguish between RSA patients and normal pregnant subjects because the downregulation of miR-103 in RSA is very sensitive, which makes miR-103 become a promising diagnostic marker and therapeutic target for RSA [64]. In addition, Ding et al. [65] discovered that M1 macrophages could also inhibit the EMT process of trophoblast cells in vitro by secreting extracellular vesicles (EVs). Through miRNA sequencing, miR-146a-5p and miR-146b-5p were identified as the highest expression of miRNAs in trophoblast cells treated with M1-EVs [65]. Further functional experiments suggested that miR-146a-5p and miR-146b-5p in EV directly inhibited TNF receptor-associated factor 6 (*TRAF6*) expression at the post-transcriptional level, which inhibited the EMT of trophoblast cells in mechanism and played an important role in the communication with trophoblast cells [65].

To summarize, the potential mechanisms of miRNAs regulating SA are varied and may involve different target genes and binding sites (Table 1). In recent years, some studies have found statistical differences in miRNAs in exosomes and plasma between patients with SA and normal pregnant subjects [8,66], which suggests that there are still broad potential pathways in which miRNAs regulate SA that we need to discover. A substantial body of evidence suggests that a miRNA–mRNA regulation network and complicated interactions and crosstalk between miRNAs are involved in the pathogenesis of SA. If this network is thoroughly investigated, it will significantly aid in the diagnosis and treatment of SA.

## 4. lncRNAs and Spontaneous Abortion

### 4.1. Overview of lncRNAs

lncRNAs were once considered to be part of transcriptional noise, but now they represent a new “style” of transcriptional and post-transcriptional gene regulation. Structurally, lncRNA is similar to mRNA, with a 5′ cap and 3′ PolyA tail structure, longer than 200 nt and has a complex secondary or tertiary structure without a highly conserved sequence [67,68]. Paradoxically, although the coding regions of lncRNAs are short or non-existent and have a low expression level in cells, they can not only be transcribed from any part of the genome but also play an imperative role in various biological activities such as cell signaling, cell differentiation, transcriptional regulation, epigenetics, and immune response [17,69,70,71,72]. lncRNAs not only regulate the expression of protein-coding genes at the transcriptional and post-transcriptional levels but also are the precursor of smaller regulatory RNAs, such as miRNAs and piwiRNAs [73]. Moreover, as one kind of ceRNA, lncRNAs can also regulate miRNA levels and mRNA stability and translation through homologous base pairing [10].

**Figure 2:**
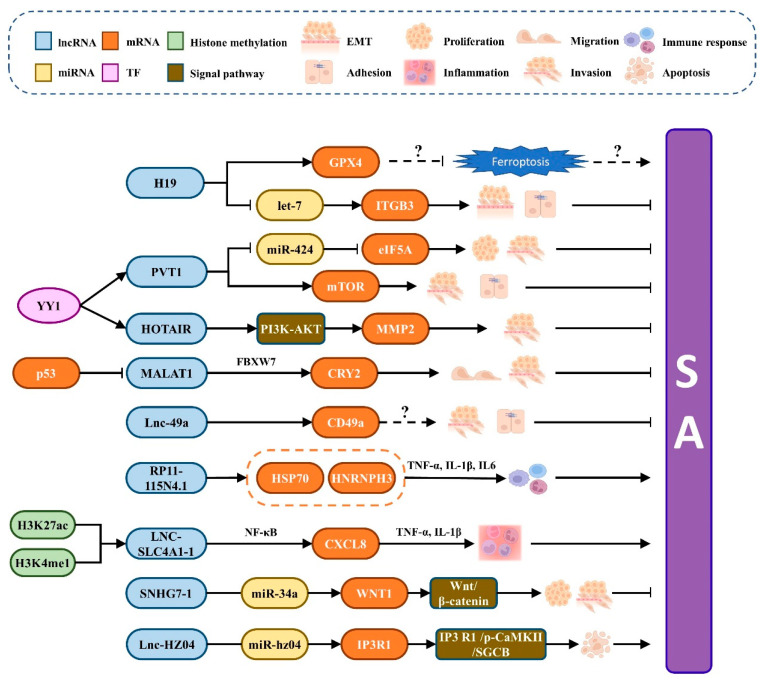
The mechanism for lncRNA to participate in SA. There are two main ways lncRNAs participate in the occurrence and development of SA: (1) directly regulate the expression of target mRNA; (2) regulate the transcription and expression of downstream target mRNA by targeting miRNAs. “?” represents our hypothesis for this regulatory pathway according to existing research.

The research on lncRNAs in SA is increasingly on the rise in recent years. Previously, researchers have identified several lncRNAs subsets that are differentially expressed in the villi of SA patients and normal pregnant women, providing evidence for the involvement of lncRNAs in the physiology and pathogenesis of SA [73,74,75,76]. These studies found that lncRNAs might be mainly involved in the pathogenesis of SA through inflammation, extracellular matrix (ECM)-receptor interaction, and apoptosis pathways, and contributed to the follow-up screening of lncRNA as a marker for the diagnosis of SA. lncRNAs are mainly involved in SA generation and regulation through two ways (Figure 2): (1) regulate the expression of target mRNA directly; (2) regulate the transcription and expression of downstream target mRNA by targeting miRNAs.

### 4.2. lncRNAs Regulate Target mRNAs Directly

Like miRNAs, lncRNAs can also regulate cellular proliferation, invasion, migration, and apoptosis by modifying target mRNAs. H19 is the first lncRNA discovered by humans, and it represents a milestone in the history of understanding and investigating ncRNA [77]. Several investigations have confirmed that *H19* is a significantly expressed gene throughout embryonic development and has a role in tumor invasion and migration [78,79]. Even though the mechanism of embryo implantation in the endometrium is similar to tumor invasion, few studies on H19 in SA have been conducted. Bai et al. [80] recently observed that the expressions of *H19* and *GPX4* in the SA group were lower than in the standard group. Silencing *H19* can downregulate the expression of *GPX4* at mRNA and protein levels in HTR8/SVneo cells. A previous study found that GPX4 is a critical regulator in the ferroptosis pathway [80]. Therefore, the correlation between the expression of *H19* and *GPX4* also implies that ferroptosis might occur in SA, opening up a new research avenue for better comprehension of the pathogenesis of SA.

Some transcription factors and genes directly regulate the expression of lncRNA, affecting its downstream regulatory pathways and controlling the occurrence of SA. Ying Yang 1 (YY1), a transcription factor with the dual role of transcriptional inhibition and activation, is a transcription factor that participates in SA by modulating the expression of cytoskeleton-related proteins to alter the invasion of trophoblast cells [81]. In RSA patients, the YY1mRNA level of primary trophoblast cells was considerably lower than healthy controls [82]. Furthermore, YY1 may directly bind to the PVT1 promoter and subsequently control apoptosis, adhesion, and invasion through the mTOR pathway, thereby affecting the invasion and adhesion of trophoblast cells [83]. Similarly, the HOX transcriptional antisense RNA HOTAIR is a YY1 target lncRNA, and the HOTAIR promoter has two binding sites that can be bound by YY1 [84]. *HOTAIR* overexpression promotes the production of matrix metalloproteinase 2 and enhances trophoblast cell invasion. Moreover, the tumor suppressor gene, *P53*, has been elucidated to regulate the expression of numerous lncRNAs [85]. Meanwhile, *P53* overexpression would reduce cellular proliferation, migration, and invasion while increasing apoptosis, resulting in SA [86,87]. Wang et al. [88] identified that the expression level of P53 protein was significantly negatively correlated with the expression level of *MALAT1* and confirmed that P53 inhibited *MALAT1* expression by directly binding to the promoter region. Subsequently, RNA pull-down and co-immunoprecipitation assays demonstrated that MALAT1 causes CRY2 ubiquitin-mediated degradation by engaging E3 ubiquitin ligase FBXW7, resulting in inhibition of SA by participating in the trophoblast cell migration and invasion [89]. Based on the above research, it is assumed that the P53-MALAT1-FBXW7-CRY2 axis plays a dominant role in the pathogenesis of SA.

Immune imbalance is one of the primary causes of SA. In decidual natural killing (DNK) cells, lncRNA has also been demonstrated to have a critical role in the pathogenesis of SA. Human DNK cells constitute the most numerous lymphocyte populations in early pregnancy at the maternal-fetal interface. They act as a regulator of maternal immunological tolerance, facilitating embryo implantation and placenta development [76]. Therefore, it is worthwhile to investigate the differential expression and regulation mechanisms of lncRNAs in the DNK cells of SA patients. Using RNA-seq technology, Li et al. [76] identified that 276 mRNAs and 67 lncRNAs were differentially expressed in the DNK of SA patients versus normal pregnant women. Based on these differentially expressed RNAs, a lncRNA-mRNA regulatory network with small-world characteristics was built, which is involved in a variety of biological processes (immune response, inflammatory response, cell adhesion, and ECM organization) and signal pathways (cytokine-cytokine receptor interaction, ECM-receptor interaction, Toll-like receptor signaling pathway, and phosphatidylinositol signaling system).

Furthermore, lnc-49a, a novel lncRNA identified recently, is a positive regulator of CD49a in DNK cells and is involved in regulating DNK cell functions, such as cellular activity, migration, and adhesion. Li et al. [90] discovered that the expression of *CD49a* was downregulated in RSA patients. In contrast, the expressions of perforin, granzyme B, and *IFNG* were upregulated, implying that lnc-49a can impede SA through an unknown mechanism by regulating the expression of the targeted gene *CD49a* [90]. In peripheral blood, some lncRNAs are related to the occurrence of SA. For example, Liu et al. [74] found that RP11-115N4.1 was the most abundant lncRNA in the peripheral blood of URSA patients. The activation of RP11-115N4.1 can significantly increase the binding protein of HSP70 and HNRNPH3, which may be one of its mechanisms for regulating immune response, and it can trigger the occurrence of SA via inhibiting the migration of trophoblast cells.

Moreover, lncRNA can influence SA through imprinting. During placental development, imprinted lncRNA has a dynamic temporal and spatial expression. Sheng et al. [91] found that the imprinted lncRNA Rian might play a crucial role in placenta development because its homologous sequence lncRNA MEG8 (RIAN) was unusually highly expressed in human SA villi. Upregulation of *MEG8* expression inhibited cell proliferation and invasion in trophoblast cell lines, whereas downregulation of *MEG8* expression had the opposite effect. These findings suggest that MEG8 plays an important regulatory role in trophoblast cell growth and function and that incorrect *MEG8* expression may result in dysfunctional trophoblast cells, which may play a role in the formation of URSA.

Furthermore, enhancer RNAs, a group of lncRNAs transcribed from enhancers, have been linked to URSA. The regulatory effect of enhancers on gene expression is a new area of study, and LNC-SLC4A1-1, transcribed from active enhancers labeled with H3K27ac and H3K4me1, has been found to bind to CXCL8 by recruiting NF-κB, resulting in CXCL8 activation. The activated CXCL8 induces an increase in TNF-α and IL-1β, intensifying trophoblast cell inflammation [92]. These studies provide new insights into understanding the role of lncRNA in pathological pregnancy and URSA research.

### 4.3. lncRNAs Regulate Target miRNAs to Regulate Their Downstream Target mRNAs

A considerable number of lncRNAs, such as ceRNA, regulate gene expression through sponging miRNA [93]. For example, H19 has been identified to compete with miRNA let-7 to control the target gene Integrinβ3 (*ITGB3*), critical in endometrial receptivity [94]. When H19 was knocked out, the expression of *ITGB3* was reduced, resulting in a downregulation of the adhesion and invasion. Further experiments have demonstrated that H19 suppresses the impact of let-7 by acting as a molecular sponge to avoid the degradation of ITGB3mRNA and improves the EVT adherence to the endometrium, thereby promoting EVT invasion during early pregnancy [94]. Moreover, PVT1 can also regulate the function of trophoblast cells through the PVT1/miR-424/eIF5A pathway [83]. When *PVT1* is overexpressed, it suppresses miR-424, which in turn decreases the production of miR-424’s target gene, *eIF5A*, favoring cell survival, proliferation, and migration [83]. Xiang et al. [95] also noticed SNHG7-1, a specific lncRNA, regulated the proliferation and invasion of trophoblast cells and promoted the occurrence of RSA via the Wnt/β-catenin signaling pathway, which was targeted by binding to miR-34a, hence encouraging the onset of RSA. Huang et al. [28] recently discovered an innovative lncRNA called lnc-HZ04, which can directly and specifically bind to miR-hz04, weakening the decreasing the effect of miR-hz04 on inositol 1,4,5-trisphosphate receptor type 1 (IP3R1) mRNA expression and stability, thus activating the IP3R1/p-CaMKII/SGCB pathway mediated by Ca^2+^ and further promoting trophoblast cell apoptosis.

It is still rare to find research on the regulation of target genes and pathways by lncRNA through sponging miRNA. Previous studies have indicated that this regulatory pathway appears to play an indispensable role in the occurrence and progression of SA (Table 2), implying a better understanding of the mechanism of lncRNA involved in SA.

## 5. circRNAs and Spontaneous Abortion

### 5.1. Overview of circRNAs

circRNAs are a new class of endogenous ncRNA, which were first discovered in 1979 [97]. However, they were subsequently regarded as “junk products” of gene expression and did not get people’s attention. Until Memczak et al. [98] and Hansen et al. [99] discovered the unique functions of circRNAs, circRNAs quickly became a new generation of star molecules in the following years. This type of RNA has a stable structure and is known for its unique closed-loop and single-stranded structure. Since there is no free end as the binding point of exonuclease, circRNA has strong resistance to exonuclease hydrolysis, which results in some unique characteristics of circRNA that other ncRNAs do not have [100,101], such as longer half-life [102]. Currently, the biological functions of circRNAs are being studied extensively. For instance, they act as miRNA sponges to regulate the function of miRNAs [103], as transcription or translation regulators to affect protein expression [98,104], and interact with proteins to regulate gene expression [105,106]. Unexpectedly, some circRNAs even have the potential to encode proteins [107]. Moreover, circRNA has been determined to be involved in the pathogenesis of many diseases, such as atherosclerosis, neurological disorders, diabetes, and cancer [101,108].

### 5.2. circRNAs and SA

circRNAs have been shown in recent studies to play a critical role in tumor regulation [109,110,111]. Whereas there are multiple similarities between tumorigenesis and embryo implantation in humans, circRNA may also play an indispensable role in cellular invasion, EMT, migration, and apoptosis. Indeed, several researchers have begun to pay attention to the correlation between circRNAs and SA (Table 2). Li et al. [112] discovered 123 significantly differentially expressed circRNAs in early RSA patients’ decidual tissues compared to normal pregnant women. Based on these significantly differentially expressed circRNAs, a miRNA-mRNA network targeted by circRNA was constructed, including three circRNAs (has_circRNA_104179, has_circRNA_103093, and has_circRNA_103092), 27 miRNAs, and 82 mRNAs. Another study analyzed the expression of circRNAs in chorionic villi of RSA patients and healthy controls by using circRNA chips and found 594 differently expressed circRNAs [113]. Further analysis revealed that the aberrant production of circRNAs in RSA villi contributes to RSA pathogenesis via sponging miRNA.

Some experiments specifically focus on the circRNA regulatory pathways in SA. Li et al. [96] investigated the effect of CIRC-ZUFSP overexpression and downregulation on trophoblast cell activity in vitro. They discovered that CIRC-ZUFSP influences the molecular mechanism of trophoblast cell migration and invasion through modulating miR-203. *STOX1* is the target gene of miR-203. Thus CIRC-ZUFSP may induce SA through the CIRC-ZUFSP/miR-203/STOX1 pathway, which inhibits the migration and invasion of trophoblast cells [96]. In addition, Zhu et al. [31] discovered that circPUM1 could promote trophoblast cell processes and anti-inflammatory effects through the miR-30a-5p/JunB axis, hence preventing the formation and progression of SA. *CircPUM1* gene knockout reduces trophoblast cell proliferation, migration, and invasion, increases trophoblast cell apoptosis, and promotes pro-apoptotic protein (cleaved caspases-3) levels and pro-inflammatory factors (TNF-α, IL-6, and IL-8) secretion, similar to the role of lncRNA CXCL8 discussed above.

Furthermore, circFOXP1 (hsa_circ_0008234), which is generated from the exon region of the *FOXP1* gene, may regulate the cell activity of many disorders, and Gao et al. [30] demonstrated for the first time that it could regulate the function of trophoblast cells via the miR-143-3p/S100A11 cascade pathway. Mechanically, circFOXP1 regulates S100 calcium binding protein A11 (*S100A11*) expression by binding to miR-143- 3p competitively. These studies have shown that circRNA has similarities to lncRNA, which can be combined with the corresponding miRNAs to regulate the expression of their downstream target genes, thereby controlling the occurrence and progression of SA. Concurrently, RNA studies have provided new ideas and research directions for the diagnosis and treatment of SA.

## 6. ceRNA Network in SA: A Systematic Perspective to Elucidate the Regulation of SA by ncRNAs

**Figure 3:**
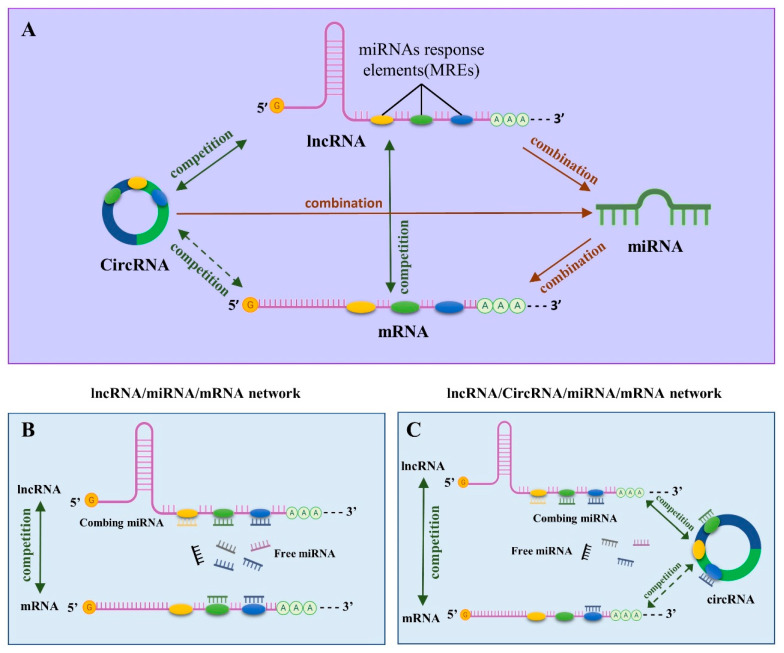
Regulatory mechanism of ceRNA network. (**A**) Relationship of mRNA, miRNA, circRNA, and lncRNA in ceRNA network hypothesis; (**B**) specific regulation mechanism of lncRNA-mediated ceRNA network. LncRNA regulates the expression of mRNA by competing for the opportunity of binding to miRNA; (**C**) specific regulation mechanism when lncRNA and circRNA are as ceRNA simultaneously. lncRNA and circRNA simultaneously compete with mRNA for the opportunity to bind to miRNA to regulate mRNA expression. LncRNA, circRNA, and mRNA compete with each other.

Based on the preceding explanation, it can be concluded that there is an intricate RNA regulatory network connected through a complex network of feedback mechanisms in the case of SA. In 2011, Salmena et al. [10] proposed the ceRNA regulatory network concept, claiming that mRNAs, transcribed pseudogenes, and lncRNA could interact via a unique “language” established by miRNA binding sites (“miRNA response elements” or “MRE”). This concept is currently being refined and is widely accepted. Non-coding RNAs, such as circRNA, were later discovered and added to this network [93]. In recent years, some studies have clarified the role of ceRNA networks in cancer development [114,115,116] and pregnancy diseases such as recurring implantation failure (RIF) [117]. More directly, Huang et al. [118] and Zang et al. [119] validated the presence of this network in SA patients in humans and pigs, respectively. However, there is a paucity of in-depth research on the role of the ceRNA network in the pathogenesis of SA. Based on the findings of previous studies of miRNA, lncRNA, and circRNA in SA, we anticipate that several ceRNA networks have the potential to serve as a guide and reference for more in-depth research of the mechanism and role of ceRNA in SA (Figure 3).

### 6.1. LncRNAs-miRNAs-mRNAs Network

According to the ceRNA hypothesis, lncRNAs operate as molecular sponges to absorb miRNAs, resulting in decreased binding to their downstream target mRNAs and restoration of mRNA activity or expression [10,93]. Huang et al. [118] and Zang et al. [119] created ceRNA networks in SA patients from humans and pigs. This network in human SA comprises 31 lncRNAs, 1 miRNA (hsa-miR-210-5p), and 3 genes (*NTNG2*, *GRIA1*, and *AQP1*) [118]. While in pigs, it consists of 4 LncRNAs (TCONS_00051274, TCONS_00161675, TCONS_00108310, TCONS_00177102), 11 miRNAs and 13 genes [119]. Some sporadic lncRNAs, such as GAS5, have also been confirmed to be correlated with miR140-5p as a ceRNA [120]. In addition, UCA has been found to operate as a ceRNA through the uptake of miR-455, which leads to the upregulation of RUNX family transcription factor 2 (*RUNX2*) expression in HTR-8/SVneo cells [121]. The H19, PVT1, SNHG7, and lnc-HZ04 described above can hypothetically be utilized as ceRNAs to absorb miRNAs and contribute to the occurrence and development of SA. However, the ceRNAs that we have identified are only the tip of the iceberg regarding the entire ceRNA regulatory network, and the comprehensive network control mechanism that underpins it is still mysterious.

### 6.2. CircRNAs-miRNAs-mRNAs Network

CircRNA contains multiple miRNA binding sites acting on miRNA sponges. These sponges work as CeRNAs to improve miRNA-induced target gene suppression by boosting their expression levels and through disease-related miRNAs interaction [122]. There are currently no relevant data to elucidate the role of the circRNA-mediated ceRNA regulatory network in the occurrence and progression of SA. Other studies, however, have shown that the circRNA-mediated ceRNA regulatory network plays a critical role in the average growth and development of embryonic organs and tissues in pig and mouse embryos [123,124]. These studies have possible implications for the universality of circRNA-mediated ceRNA networks in various organisms.

Similarly, some researchers have already discovered circRNA-mediated ceRNA networks in pregnancy diseases, such as preeclampsia (PE) and RIF [125,126]. Contemporary research on the role of circRNA in SA has recently shown that circRNAs regulate SA through the circRNA-miRNA-mRNA axis. Based on a solid body of evidence, we have reason to believe that the circRNA-miRNA-mRNA network is essential in the occurrence and regulation of SA, which suggests that we should focus our efforts in the future on ceRNA to reveal the mechanism of circRNA in SA.

### 6.3. lncRNAs/circRNAs-miRNAs-mRNAs Network

Both lncRNA and circRNA contain miRNA binding sites and bind the same miRNA. These miRNA binding sites exist in a competitive interaction, complicating the connection and regulation mechanism of this ceRNA network. As a result, searching for such a ceRNA network necessitates the use of more advanced and mature RNA-seq technology and bioinformatics analysis methods. Several researchers have discovered that ceRNA networks mediated by lncRNA and circRNA exist in post-menopausal complications and PE [127,128]. These networks are usually more extensive and complex than the single lncRNA-mediated or circRNA-mediated ceRNA networks.

Based on the above research results, we believe that there are at least one or more ceRNA regulatory networks in SA, and these networks have critical regulatory effects on the onset and progression of SA. Identifying these networks and RNA markers based on them is crucial in understanding the specific etiology diagnosis and treatment of SA.

## 7. Conclusions and Perspective

This study discusses the most recent advances in ncRNAs research, the ceRNA regulatory network, and potential molecular pathways relevant to the incidence and progression of SA. Through these studies, we have established that a complex ceRNA regulatory mechanism exists in the organism that regulates cell proliferation, invasion, migration, EMT, apoptosis, and angiogenesis to regulate the occurrence and development of SA. With advancements in technology such as next-generation and the maturation of RNA chips, ncRNAs with differential expression will be identified in SA. These ncRNAs will provide reference and insights into the construction of ceRNA networks and the in-depth study of the regulation of differentially expressed ncRNAs in SA.

SA in pigs is a major problem that impairs the economic efficiency of commercial pig production. Given the highly conservative nature of some ncRNAs, such as some miRNAs and circRNAs, studies of ncRNAs in humans and mice may provide valuable references for researching the regulatory mechanisms of ncRNAs in porcine SA occurrence. Likewise, owing to the similarity with humans in size, physiology, and genomic characteristics, pigs are generally considered to be the best animal model for researching human diseases [129], which addresses the part of the research on SA that is difficult to conduct in humans due to the ethical problem. This can provide a deeper understanding for researchers of the occurrence mechanism of SA and provide a theoretical basis for treating and preventing SA.

Moreover, ncRNAs are becoming increasingly important in biomedical research as new biomarkers in the diagnosis, prediction, prognosis, and therapeutic response to diseases [130]. ncRNA expression profiles are more effective than mRNA expression profiles in distinguishing normal and pathological tissues [131]. Therefore, searching for the differentially expressed ncRNAs in SA will not only provide us with a more comprehensive understanding of the pathogenesis of URSA but also enable the selection of certain ncRNAs as early predictors of SA, which will improve the prevention and treatment of the disease.

Although recent studies have partially explained the role and mechanism of ncRNAs in SA, we still lack a comprehensive understanding of this process, and there are still some challenges and mechanisms of ncRNAs in SA that need to be resolved and explored. Undoubtfully, the pathogenesis and regulatory network of ncRNA on SA will be elucidated with an in-depth study of ncRNA, which will also accelerate the process of the quest for potential RNA markers for SA diagnosis and treatment. These regulatory networks have potential applications in obstetric practice and pork production, improving women’s health and commercial pig production efficiency.

## Figures and Tables

**Table 1:** miRNAs involved in regulating SA genesis and development.

miRNA Names	Expression in SA	Target	Tissue	Function	Reference
MiR-93	Upregulation	*BCL2L2*	Chorionic villi, trophoblast	Promotes proliferation, migration, invasion, and apoptosis	[40]
MiR-19b	Upregulation	*PTEN*	Placental villi	Regulates abnormal cellular invasion and apoptosis	[41]
MiR-494	Downregulation	*PTEN*	Placental villi	Regulates abnormal cellular invasion and apoptosis	[41]
MiR-365	Upregulation	*SGK1*	Decidua	Regulates cellular apoptosis	[43]
MiR-199B-5p	Upregulation	*SGK1*	Decidua	Regulates cellular apoptosis	[44]
MiR-520	Upregulation	*PARP1*	Trophoblast	Promotes DNA damage-induced apoptosis	[45]
MiR-27A-3p	Upregulation	*USP25*	Trophoblast	Regulates the process of EMT, cell invasion, and migration	[48]
MiR-16	Upregulation	*VEGF*	Placenta	Regulates angiogenesis and development	[49]
MiR-219a	Downregulation	*VEGFR2*	Trophoblast	Negative regulation of cellular proliferation and invasion	[26]
MiR-155-5p	Downregulation	NF-kB pathway	Decidual stroma	Promotes cellular growth and proliferation and inhibits apoptosis	[25]
MiR-34a-3p/5p	Upregulation	*P53*	NK cells	Regulates cellular apoptosis and proliferation	[57,58]
MiR-141-3p/5p	Upregulation	*PTEN*	NK cells	Regulates cellular apoptosis and proliferation	[57,61]
MiR-24	Downregulation	*IFNG*	NK cells	Regulates cellular apoptosis and proliferation	[57,59,60]
Hsa-miR-5787	Upregulation	*EIF5*	NK cells	Inhibits cellular growth	[62,63]
MiR-103	Downregulation	STAT1/IRF1 pathway	M1 macrophages	Negative regulation of cellular polarization	[64]
MiR-146a/b-5p	Upregulation	*TRAF6*	M1 macrophages	Inhibits EMT and maintains cellular interaction	[65]

**Table 2:** lncRNAs and circRNAs involved in the occurrence of SA.

RNA Names	Expression in SA	Target	Tissue	Function	Reference
H19	Downregulation	*GPX4*	Placental villi	Regulates invasion and migration, and may be related to ferroptosis.	[80]
		Let-7		Competes with miRNA let-7 to control the target gene *ITGB3*	[93,94]
PVT1	Downregulation	MTOR	Trophoblast	Control apoptosis, adhesion, and invasion through the mTOR pathway.	[82,83]
		MiR-424		Regulate the function of trophoblast cells through the PVT1/miR-424/eIF5A pathway	[83]
HOTAIR	Downregulation	PI3K-AKT	Trophoblast	Promotes the production of matrix metalloproteinase 2 and enhances cell invasion	[84,85]
MALAT1	Downregulation	*FBXW7*	Trophoblast	Inhibits SA by playing a key role in the P53-MALAT1-FBXW7-CRY2 axis.	[88,89]
Lnc-49a	Downregulation	*CD49a*	DNK cells	Impedes SA by regulating the expression of CD49a	[90]
RP11-115N4.1	Upregulation	*PARP1*	Peripheral blood	Inhibits the migration of trophoblast cells by increasing HSP70 and HNRNPH3.	[74]
MEG8	Upregulation	Unknown	Chorion villi	Inhibits cell proliferation and invasion in trophoblast cell lines	[91]
LNC-SLC4A1-1	Upregulation	*CXCL8*	URSA Villi	Intensifies trophoblast cell inflammation by activating CXCL8	[92]
SNHG7-1	Upregulation	MiR-34a	Placental villi	Promotes the occur-rence of RSA via the Wnt/β-catenin signaling pathway	[95]
Lnc-HZ04	Upregulation	MiR-hz04	Villi	Weakens the decreasing effect of miR-hz04 on inositol IP3R1 expression and stability	[28]
CIRC-ZUFSP	Upregulation	MiR-203	Trophoblast	Inhibits migration and invasion of trophoblast cells through the CIRC-ZUFSP/miR-203/STOX1 pathway	[96]
CIRCPUM1	Downregulation	MiR-30a-5p	Placenta	Promotes trophoblast cell processes and anti-inflammatory effects through the miR-30a-5p/JunB axis	[31]
CircFOXP1	Downregulation	MiR-143-3p	Trophoblast	Regulates the function of trophoblast cells via the miR-143-3p/S100A11 pathway	[30]
